# Fiber-Optic Sensors (FOS) for Smart High Voltage Composite Cables—Numerical Simulation of Multi-Parameter Bending Effects Generated by Irregular Seabed Topography

**DOI:** 10.3390/s22207899

**Published:** 2022-10-17

**Authors:** Monssef Drissi-Habti, Abhijit Neginhal, Sriharsha Manepalli, Valter Carvelli

**Affiliations:** 1 COSYS Department, Université Gustave Eiffel, F-77447 Marne-la-Vallée, France; 2 Department A.B.C., Politecnico di Milano, Piazza Leonardo Da Vinci 32, 20133 Milan, Italy

**Keywords:** structural health monitoring, fiber-optic sensor (FOS), multi-axial strains, smart energy transport cable, smart composite, numerical simulation

## Abstract

Offshore renewable energy requires reliable high-voltage electric power cables to transport electricity to onshore stations. These power cables are critical infrastructures that are shipped to deep seas through shipping and handling operations and, once mounted, must then evolve in extreme conditions (sea, salt, wind, water-pressure, seabed topography, etc.). All of these operations and working conditions can lead to yielding of copper conductors, often resulting in electric shutdown. Indeed, copper is an excellent electric conductor (conductivity), but its mechanical properties are very poor. If any negligence occurs during the shipping and/or handling operations, copper can undergo plasticity, with effects on both mechanical and electric properties. It is therefore of prime importance to establish a reliable structural health-monitoring (SHM) technique that will enable the continuous recording of copper strain and temperature along a cable, and this has been proven using fiber-optic (FOS) sensors, when the phase is under tensile loading. In this prospective article, the scope is to maintain previous simulations and thus show that by the judicious placement of FOS, one can monitor strain and temperature within cables that are submitted to a bending. This article does not aim to deal directly with the case of a cable that undergoes bending on sloppy areas in seabeds. The idea behind the work is to suggest a concept for the use of embedded fiber-optic sensors and to think about all of what remains to be done as research in order to further suggest this technology to cable manufacturers.

## 1. Introduction

Carbon neutrality passes, among others, through the development of reliable renewable energy sources, among which is offshore wind energy. At the European level, offshore wind-production is expected to range from 230 to 450 GW, by 2050, to target climate neutrality. This ambition is corroborated by the International Energy Agency (IEA), who expect offshore wind energy to be ranked as the number one source of electric power in Europe within less than twenty years. Scaling up from 20 GW today to 450 GW by 2050 will require a revolutionary approach. Indeed, it is clear that the carbon neutrality economy needs renewable energy sources and associated technologies to be developed at a huge scale, which imposes passes through upgrading and large development of infrastructures (both existing and new ones). A lot of research has been devoted to offshore energy structures in recent years, especially on large-dimension wind blades and high-voltage electrical transport cables [1,2,3,4,5,6,7,8,9]. The world is changing, and so are its energy needs. In an attempt to combat the effects of climate change, countries are investing in a wide variety of renewable energy sources. One of the fastest-growing energy solutions for coastal and island countries is offshore wind energy. These offshore grids provide reliable renewable energy at a cheaper rate without being detrimental to the environment, compared to the conventional resources. With various grid structures and networks, the maintenance of these structures is also a major concern [10]. High-voltage electric cables for offshore wind energy are critical structures that have to perform in a reliable way (1) when carrying electric power ashore. It has been shown that shipping, handling, and embedding in bed seas high-voltage cables can result in an upfront performance loss, which may add to the constraint of use in very aggressive media such as seas and thus to a fear of premature shutdown. Shutdown means not only drastic budget losses but also produce significant problems that can, geo-politically, have serious consequences. Apart from the working and maintenance expenses, offshore cables are prone to failures which account for 80% of total financial losses and insurance claims. In the past 7 years, about 90 offshore cable failures have been reported, with over €350 million in insurance claims. According to [11], the repair costs of an offshore cable can be between €0.7 million and €1.5 million, which is very expensive. The loss of performance of cables is mainly due to the plastic deformation of copper, which occurs with very small loads and directly impacts other physical properties. The above proves beyond any doubt that it is essential to follow live and very precisely what is happening inside high-voltage cables for offshore farms, through the insertion of sensors of several kinds in their vicinity, which will make it possible to closely monitor the levels of deformation and temperature of the copper in service, for example, by using fiber-optic sensors (FOS).

**Figure 1:**
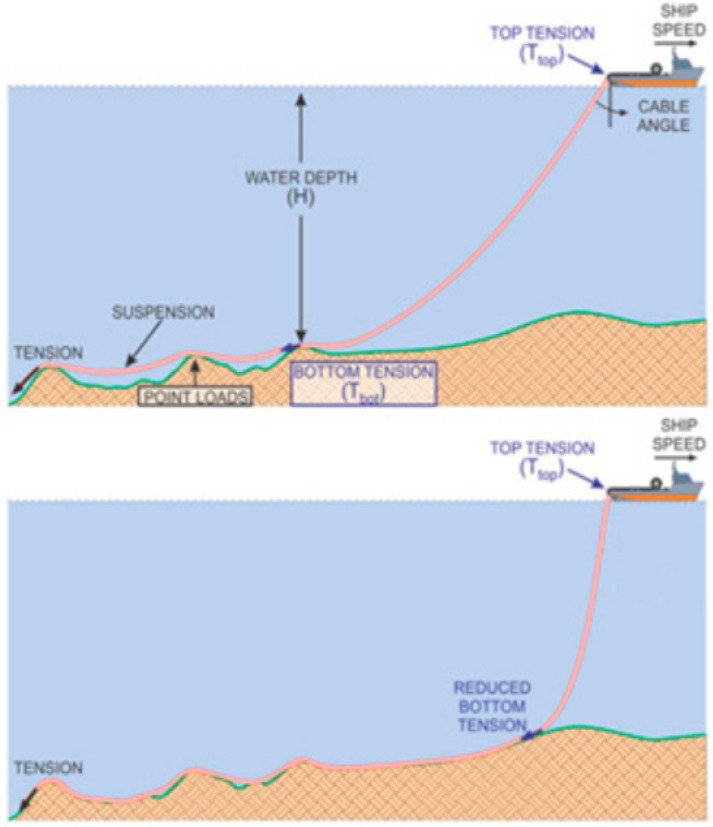
An example of the steep and sloppy topography at the bottom sea [25].

In a previous article [1], structural health monitoring (SHM) using the optimal placement of FOS sensors inside a cable phase was proposed and demonstrated promising results, in the case where the cables are stressed in tension. In this new prospective article, numerical simulations were carried out in order to conceptualize the placement of FOS this time under bending stress. This mode of solicitation must absolutely be studied insofar as the seabed has topography that is anything but flat. It is therefore more than likely that in many places where the sea bottom is sloppy and steep or even under the effect of currents, the cable may be positioned in a zone where locally the bending is important, even too important (Figure 1). For example, in order to stiffen umbilical high-voltage cables against the effects of excessive curvatures, stiffeners are implemented [12,13,14,15]. High-voltage cables of the umbilical type are connected to devices, whether a wave energy device, offshore stations, or a connection box. The external stresses applied on umbilical cables are significant and can lead to damage generated by excessive bending and/or by fatigue. In order to counteract these consequences, bending stiffeners are implemented, which locally add resistance to bending and the curvatures generated. There are static bending stiffeners that are used in particular for protection against bending during installation. These stiffeners are generally made from molded polyurethane.

From what precedes, it is crucial to have a precise idea of the impact of this bending on the cable, by using FOS as an SHM technique [16,17,18]. It is worthwhile to note that significant bending will lead to plastic deformation of copper and will cause a temperature increase; accordingly, it is important to know that the temperature increase must in no case exceed 90 degrees (fear of electric shutdown).

Looking at the current cable design, there is no possible room closer to the conductor in which the FOS could be embedded for monitoring purpose. Placing the FOS inside the XPLE (cross-linked polyethylene) insulator entails placing the FOS far from the copper core, and this may account for inaccurate data, due to both a major offset and the presence of other materials. Adding a separately dedicated material to the host FOS endowed with protection will increase the diameter of the cable, which will be financially very expensive when envisaging thousands of kilometers of submarine power cables.

Currently, XLPE is the top insulator that is used in high-voltage electric cables, thanks to its remarkable and reliable properties (both electrical and thermo-mechanical) associated with its very competitive cost [19]. The XPLE insulator can keep its properties safe up to a working temperature as high as 90 °C, while its overload temperature of approximately 105 °C is still bearable [20,21]. In the event of one or more copper wires issues (individual failure and/or plastical deformation), this leads to a core overheat at the section where failure/deformation occurs [9]. Hence, once the core temperature rises above 90 °C, the whole electric system can experience shutdown. It is worthwhile to note that high-temperature working conditions can also accelerate the aging of XPLE, which has a serious influence on cable service life [22]. Cable efficiency is a direct function of the operating temperature, which means that as long as the service temperature is much lower than 90 °C, the cable will be preserved and its life will be longer [23]. The conductor temperature has a direct influence on XPLE aging, thus showing how critical the real-time continuous monitoring of the conductor temperature [24] is. The monitoring of stress and temperature through embedded sensors is therefore mandatory.

The embedding of optical sensors within the XPLE or in-between copper wires is technologically challenging due to the fear of breaking the sensors . This fear still exists whatever the area of placement of FOS (embedded along the central copper wire and/or helical-wound the same way as other copper wires). Indeed, extensive inter-copper wire sliding/friction can arise with a high failure probability of breaking FOS. The option of embedding FOS within thermoplastic materials (insulator XPLE) can be an alternative option, though it would lead to the underestimation of the magnitude of the thermal parameters of the cables. Whether the error is constant or increasing, when shifting the positioning of the FOS between the copper section and the edge of the isulator one, is a key-question that must be answered properly.

In this article, a solution of embedding FOS in-between copper wires or within the insulator XLPE (cross-linked polyethylene) is suggested. The placement strategy is targeting the fine bending monitoring of phases. The points listed in the introduction are analyzed numerically as prospective work, which may help other engineers to target the right cost-effective solutions. The work shows that the concept of placement, previously introduced in the case of tension, is still relevant for monitoring bending.

## 2. Geomteric Model

**Figure 2:**
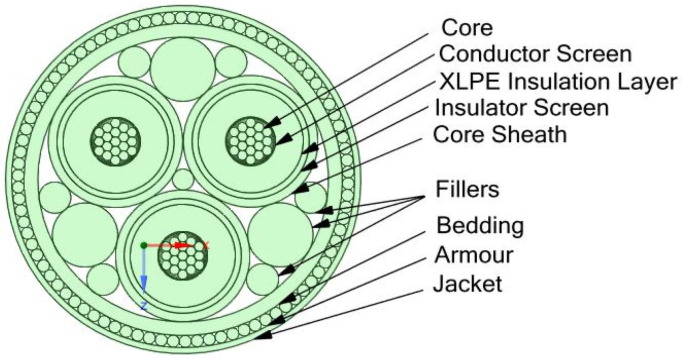
Cross-section of the cable [1].

**Figure 3:**
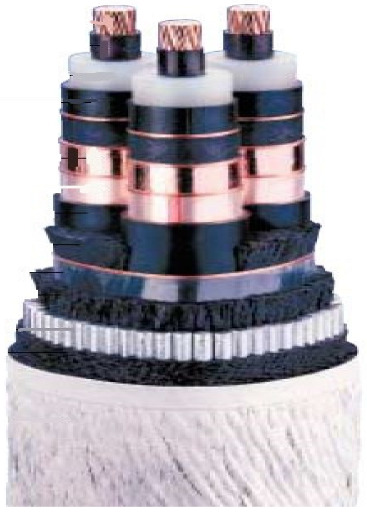
Diagram of the cable [1].

In the model that is suggested, a single phase of the 35kV Nexans submarine power cable is considered (Figure 2 and Figure 3, Table 1), 2XS2YRAA 18/30(36) kV [1] . Owing to its high- and low-temperature performance, XPLE is recommended for high-voltage electric cables [26]. The Young’s modulus of XPLE decreases with rising temperature, while its thermal expansion shows reverse behavior (the thermal expansion coefficient increases in the temperature range 25 °C to 105 °C, by 5%, while the copper expands by less than 3% in the same temperature range [27]).

## 3. Mechanical Boundary Conditions

**Figure 4:**
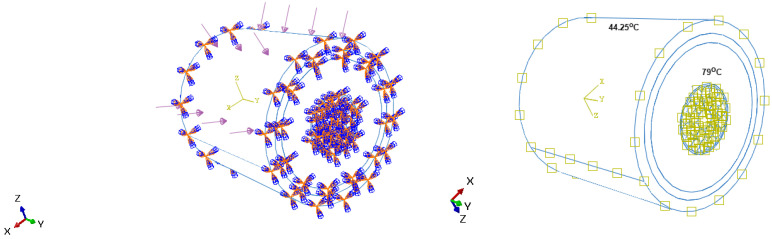
Mechanical and thermal boundary conditions considered in the research.

As previously written, high-voltage cables are designed to withstand complex loading that is caused by various operations of manufacturing, handling, and setting operations in bed seas. In the case of inappropriate design and layouts, the cable is more prone to damage, which can result in heavy and costly maintenance. In this article, a pure bending loading was assumed, which is not reflected in the reality. The idea here is to have the basic outputs for further research. The bending loading is used as a local loading and is chosen because the topography of the seabed is absolutely non-uniform. Many rocks, submarine canyons, and trenches can cross the pathway of the cables, resulting in bending. The flexural loading is detrimental as it can lead to the plasticity of the copper and/or the failure of the wires. Numerical simulations have been carried out using Abaqus software. Bending loading is considered applied on the bottom face of the submarine cable as shown in the Figure 4. As detailed in a previous article [1], the same assumptions have been considered, i.e., the bending loading is applied along the lateral axis of the submarine cable with a magnitude of 80 MPa (it is assumed that the cable is submitted to hydrostatic pressure at a depth of 8000 m), and both the axial ends of the submarine cable are constrained with all the degrees of freedom fixed.

## 4. Thermal Boundary Condition

For calculating the temperature and the ampacity of the submarine cable, the equivalent thermal resistance method is considered with reference to the International Standard IEC 60287 [28], knowing that the IEC cannot consider air convection and radiation coupling. This work is concerned with conduction as a transfer of heat only and thus neglects other aspects, and as a matter of fact the IEC 60287 Standard can be used. The details of the IEC 60287 and associated treatments that are developed in this article have been developed elsewhere [9]. In this article [9], it has been considered that sea water with a temperature of 15 °C is the medium in which the cables are immersed. The thermal analysis of cables targets the estimate of the temperature increase in the cables, caused by the heat of the conductor and by the flow circulating radially to the ambient (heat sink) . This involves various materials. The heat transfer is considered to take place in a steady-state phase, which assumes the analysis is time-independent.

## 5. Results and Analysis

The locations chosen for FOS-sensor embedding in the phase (middle, helical-wound around the wires or even in XPLE, at various distances from the conductor) have been discussed in detail elsewhere [1]. Test configurations that were considered were FOS linear placement along the wires and/or within XPLE, and helical-wound around copper wires. It should be emphasized that the current article is prospective because regarding the conceptualization of FOS embedding in electric phases, it is far from industry application.

### 5.1. Test Case A—Positioning of Linear FOS in Parallel Positioning with Varying Length from the Core

**Figure 5:**
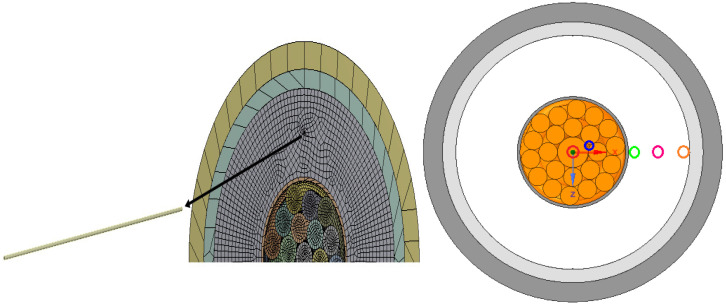
Arrangement of FOS over various different locations within the cable.

**Figure 6:**
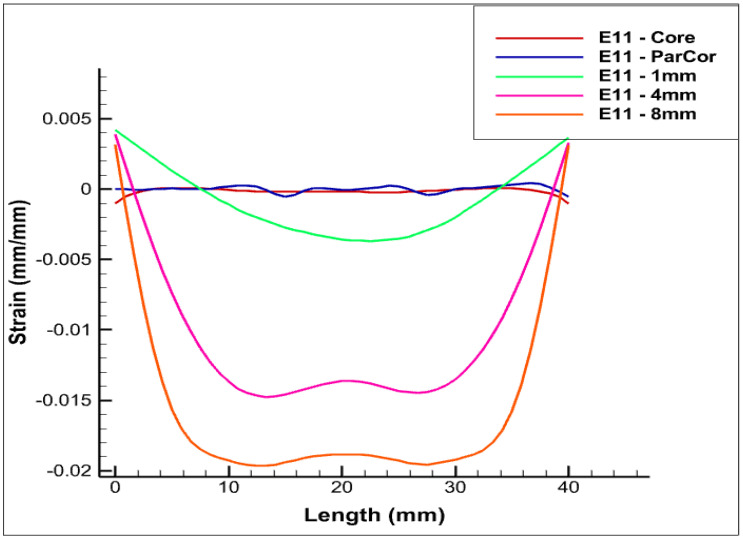
Exx for various positionings of FOS in the case of the pure bending test case, along *x* axis.

The various positionings of FOS within the phase are shown in Figure 5 and Figure 6. The case that is set at a distance of 4 cm within XPLE insulator is depicted in Figure 6.

In Figure 5, different colors of linear-placed FOS are shown. Blue refers to FOS placed parallel to the central wire inside copper. The other colors (pink, green, and orange) are associated with the embedding of FOS within XPLE at various distances from copper.

It is clear that fiber-optic sensors that are placed in contact with copper will show the same deformation as that of the central wire. This is clearly shown when comparing (Figure 6) the strain values of the central wire (in red) with the ones corresponding to the FOS parallel and in contact with copper (in blue). Although the positioning parallel to and in contact with the central copper wire seems technically the most convenient to obtain the true strain deformation of copper, the solution is not straightforward from an engineering point of view. Indeed, the solution comes with a high probability of FOS failure, due to the extensive sliding/friction, which is expected while the cable is in bed seas.

**Figure 7:**
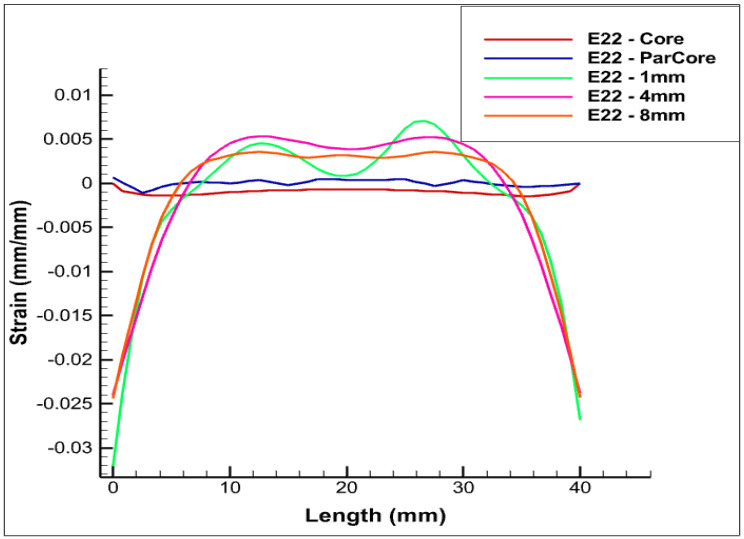
$E_{yy}$ Eyy for various positionings of FOS in the case of the pure bending test case, along *y* axis.

**Figure 8:**
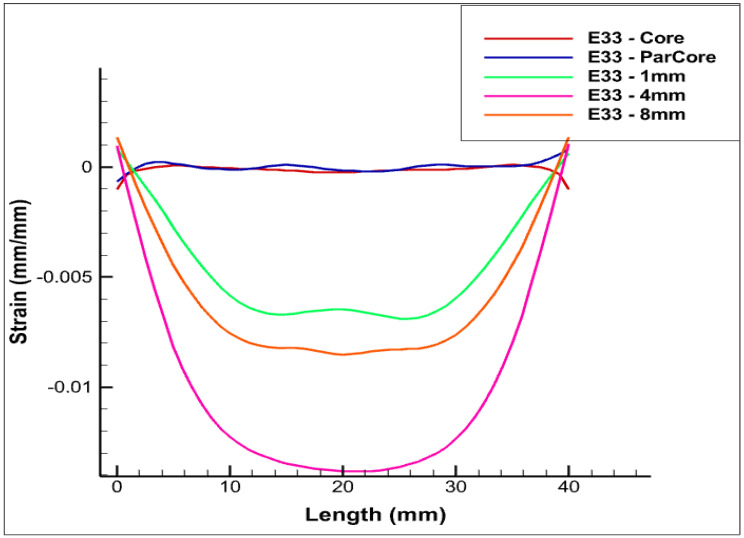
$E_{zz}$ Ezz for various positionings of FOS in the case of Pure Bending Test Case, along *z* axis.

A slight offset is pictured in the above Figure 6, Figure 7 and Figure 8 that corresponds to the linear-placed FOS, located at 1 mm within the insulator (in green). The boundary condition applied explains the extreme strain values shown at half a length of the phase. The constraints on both sides of the cables produce strain values around zero. As explained above, the placement of FOS was considered further within the insulator (4 mm, in pink). The strain values increase as function of the distance from the core. Strangely, the $E_{zz}$ strain magnitude obtained is greater than all of the cases considered. However, this could be rectified as the strain value is in the magnitude of $e^{-2}$, and this may enable us to consider this placement as a potential one that could come with credible copper wire strain values. The last case of positioning considered is that which places the sensor at 8 mm, in the insulation (orange), which corresponds to a distance of 15 mm from the central copper wire. This positioning delivers copper deformation values that are in a major shift. As a result, this positioning is completely irrelevant.

As seen in the previous paragraph, the positioning of the fiber-optic sensors inside the central copper wire delivers the most realistic copper-strain values. There nevertheless remains the problem of the technical implementation of this solution, which is doubtless very difficult. Indeed, the coating of the sensors must be very resistant and very reliable against friction with copper. The same risk should still be minimal when the sensors are helical-wrapped around the wires. This last positioning solution seems scientifically ideal insofar as it would provide multi-parameter deformation of copper. The case that corresponds to the positioning of the sensors at 1 mm in the insulation is interesting and leads to a lateral constraint ($E_{xx}$) endowed with a minimal difference compared to that in the center. The difference is still minimal when considering the longitudinal deformation. When compared to the other two cases, there is a difference in longitudinal deformation. Finally, when considering both the deformation measurements and the risks associated with the positioning within copper wires, the positioning of the sensors at 1 mm within the insulation (green) seems to be a solution that combines precision, reliability, and fiber-optic monitoring security.

### 5.2. Test Case B: Placement of FOS Helical Wound around the Complete Core

**Figure 9:**
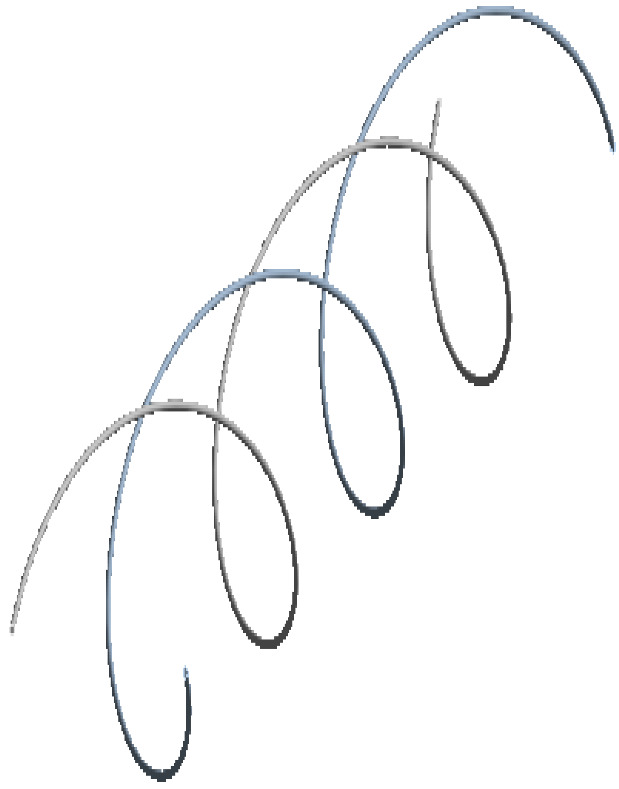
Helical arrangement FOS.

**Figure 10:**
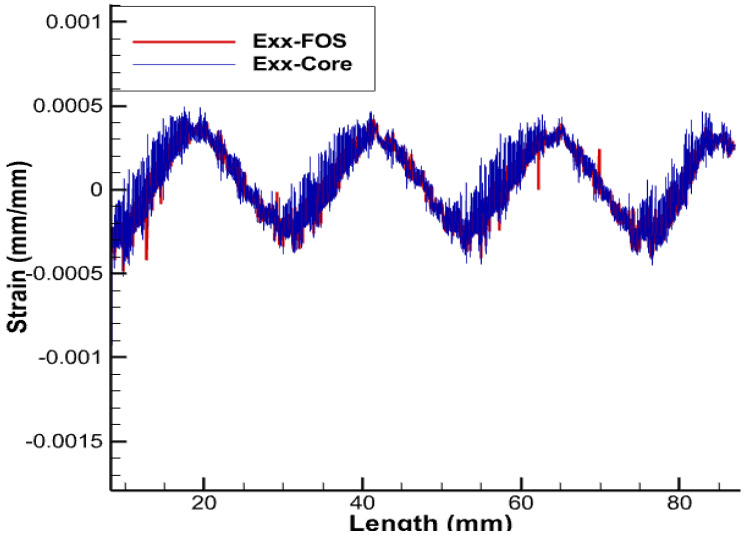
$E_{xx}$ for helical arrangement of FOS.

**Figure 11:**
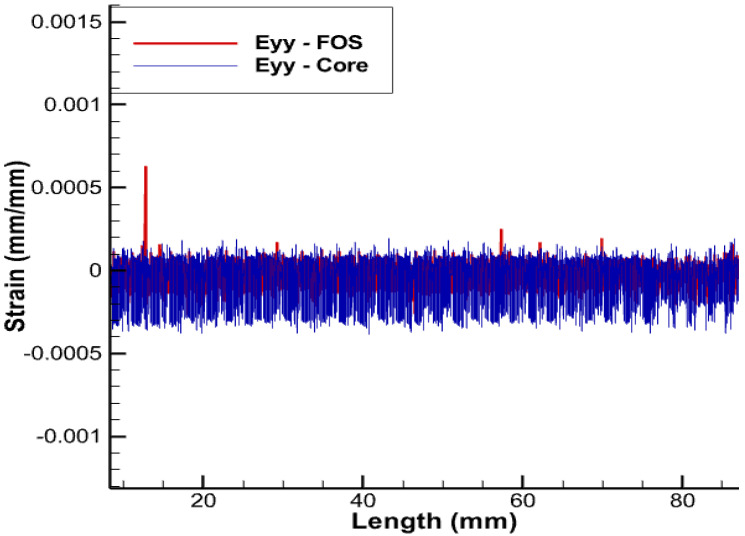
$E_{yy}$ for helical arrangement of FOS.

**Figure 12:**
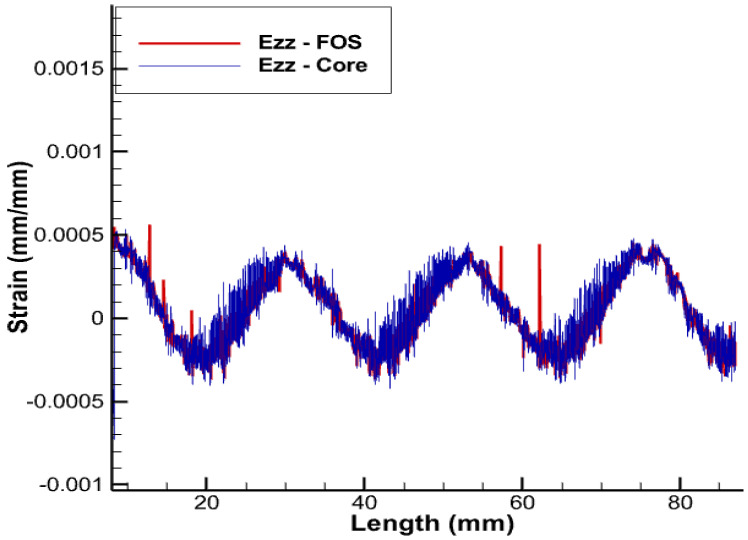
$E_{zz}$ for helical arrangement of FOS.

Getting complete information about deformation of copper wires and thus knowing precisely the level of stress achieved and eventually whether yielding and/or plasticity are in progress and/or mastered is only achieved if the positioning of sensors exactly matches the geometry of copper wires to be monitored. It should still be remembered that the plasticity of wires limits electric and thermal conduction, which comes with a real fear of shutdown. All wires but the central core are helical-wound; it is therefore clear that the positioning sensors’ helical-wound wires are naturally the best since they will provide access to the complete pieces of information about copper strains in the three dimensions (strains in linear, shear, and lateral directions), which is a significant advantage over linearly-positioned FOS, which just provides the lateral strain values without any information on longitudinal and shear loads [3]. Obviously, the positioning helical-wound at the contact area between the copper and XPLE comes with a strong challenge of protecting sensors from excess sliding/friction. The results that are depicted in the Figure 9, Figure 10, Figure 11 and Figure 12 are obtained with a pitch size the same as that of the copper coil, i.e., the pitch of the helical wire used is half of the total axial length.

The deformations corresponding to fiber-optic sensors positioned helically around the outermost copper wires are shown in Figure 9, Figure 10, Figure 11 and Figure 12. Considering the strain values, the trend in both cases is almost identical. As for an engineering solution that can tackle sliding/friction issues, one of the possible solutions is the one that uses polymer-derived fiber-optics and/or friction-resistant fiber-optic coatings along with other options . Some articles are available that address the SHM of structures with embedded optical fibers [29].

## 6. Conclusions and Discussion

Embedding FOS as an SHM technique within a high-voltage phase of a smart electric cable for offshore can be a reliable way of continuously monitoring those critical structures. The monitoring can be done remotely, and this is a key-point advantage. This concept was previously introduced in the case of the tensile loading of the cable. The current article focuses on bending aspects that are critical, given the topography of sea beds that are often steep. The numerical model is able to picture clearly various scenarios of embedded FOS and associated monitoring, thus hopefully helping engineers with future cable design. Both strain values of FOS and copper wires were studied and compared, under bending, which can simulate the irregular topography of sea beds. The linear placement of FOS parallel to the central copper wire naturally produces the best results, but these results cannot screen the fact that engineering issues pertaining to extensive FOS-copper friction may lead to the sensor failure unless appropriate FOS coating technologies are applied. Other embedding locations show promising results, too, among which is is the embedding of FOS at a distance of 1 mm inside the XLPE. This location is by far the most secured one given that it comes with no fear of FOS failure, added to technical embedding easiness. This option can be further assessed to provide for the most reliable SHM for smart-energy transport cables. Ideally, the option of helical-wound FOS embedding in contact with copper wire is by far the most informative, given the possibility to record lateral, shear, and longitudinal strains. Here again, the engineering challenge lays in the development of appropriate and resilient FOS coating. Challenges and applications of SHM-based optical fibers have been addressed in numerous articles, among which is the case of aeronautics [29].

If one wants to brain-storm about a reliable technological solution capable of protecting fiber-optic sensors embedded within copper wires, the one that comes to mind spontaneously is the case where optical fibers sensors are protected by a copper coating, which can be used at low and high temperatures and for which a fine carbon inter-phase is added in order to improve both the hermetic and mechanical properties of the sensors [30]. This system has already demonstrated proof of reliability in bio-medicine, the oil industry, and aeronautics, for temperatures ranging from −196 °C to +600 °C (more than adequately covering the fields of use of high-voltage electrical cables) [31].

## Figures and Tables

**Table 1:** Material table assigned to single-phase cable [1].

Materials	Density (kg/m^3^	Young’s Modulus (MPa)	Poisson’s Ratio	Thermal Conductivity (W/mK)
Copper	8300	1.1 $\times \;10^{5}$	0.3	370
Semi-Conducting Polymer	1000	1500	0.4	0.1
XLPE	955	1250	0.4	0.28
Polyethelene Black	958	1050	0.4	0.2
Acrylate	950	2700	0.35	0.2
Polymide	1100	3000	0.42	0.8

## Data Availability

The availability is total.
